# Penetration Depth Prediction of Infinity Shaped Laser Scanning Welding Based on Latin Hypercube Sampling and the Neuroevolution of Augmenting Topologies

**DOI:** 10.3390/ma14205984

**Published:** 2021-10-12

**Authors:** Yisheng Yin, Chengrui Zhang, Tieshuang Zhu

**Affiliations:** 1 Key Laboratory of High Efficiency and Clean Mechanical Manufacture (Ministry of Education), School of Mechanical Engineering, Shandong University, Jinan 250061, China; 201713802@mail.sdu.edu.cn (Y.Y.); 201913957@mail.sdu.edu.cn (T.Z.); 2 National Demonstration Center for Experimental Mechanical Engineering Education, Shandong University, Jinan 250061, China

**Keywords:** infinity shaped laser scanning welding, laser beam oscillation, penetration depth prediction, Latin hypercube sampling (LHS), neuroevolution of augmenting topologies (NEAT)

## Abstract

This paper builds an infinity shaped (“∞”-shaped) laser scanning welding test platform based on a self-developed motion controller and galvanometer scanner control gateway, takes the autogenous bead-on-plate welding of 304SS with 3 mm thick specimens as the experimental objects, designs the experimental parameters by the Latin hypercube sampling method for obtaining different penetration depth welded joints, and presents a methodology based on the neuroevolution of augmenting topologies for predicting the penetration depth of “∞”-shaped laser scanning welding. Laser power, welding speed, scanning frequency, and scanning amplitude are set as the input parameters of the model, and welding depth (WD) as the output parameter of the model. The model can accurately reflect the nonlinear relationship between the main welding parameters and WD by validation. Moreover, the normalized root mean square error (NRMSE) of the welding depth is about 6.2%. On the whole, the proposed methodology and model can be employed for guiding the actual work in the main process parameters’ preliminary selection and lay the foundation for the study of penetration morphology control of “∞”-shaped laser scanning welding.

## 1. Introduction

A commonly used steel grade is 304 stainless steel (304SS). The addition of alloy elements such as Cr and Ni endows 304SS with good processing performance and corrosion resistance in most harsh environments. This steel grade is widely used in the aviation, aerospace, shipbuilding, medical, and automotive fields. During its application, welding from the front when it is difficult to turn over or inconvenient to weld inside is inevitable for the assembly of components and the quality of welded joints plays a vital role in deciding the reliability and satisfactory property of the fabrications; furthermore, penetration morphology has an important influence on the weld quality [1,2].

Laser welding is considered a preferable method to achieve the joining of medium plates, given its advantages of high power density, low heat input, deep penetration, narrow heat affected zone, and excellent mechanical properties [3,4]. However, some weld defects such as underfill, crack, and porosity easily occur in laser welding [5,6].

Laser scanning welding is a new welding technique that emerged in recent years. Different scanning welding trajectories can be achieved by controlling the galvanometer scanner [7]. Laser scanning welding technology reportedly has the potential to improve tolerances of joint gaps, microstructure homogeneity, and weld quality. Kraetzsch et al. found that the cracks of dissimilar Al/Cu and Al/Ti welds could be reduced by beam oscillation [8]. Berend et al. confirmed that high frequency oscillation could eliminate the “humping” defect of Al alloy weld [9]. Vänskä et al. found that the assembling misalignment of 5 mm-thick stainless steel tube can be overcome by using beam oscillation to widen the weld [10]. Wang et al. also developed circular oscillation welding, obtained sound welds, and established that the beam oscillation was responsible for an increase in the weld ductility [11]. Zhou et al. studied the influence of the scanning trajectory, scanning amplitude, and scanning frequency of a beam during laser scanning welding on the pores of a 6061 aluminum alloy and effectively inhibited the generation of pores [12]. Yang examined the welding of aluminum alloy under laser scanning tracks with “—”, “|”, and “O”-shapes, thereby reducing the weld porosity [13]. “∞”-shaped laser scanning welding has gained significant interest from academia and industry in recent years. Moreover, the “∞”-shaped laser scanning welding pool has good stability and an obvious inhibitory effect on the pore defects in the aluminum alloy weld, thus reducing the porosity to within 1% [7]. Wang et al. verified that compared to the linear and circle oscillating modes, the novel infinity beam oscillating presented fewer welding defects and better mechanical properties [14]. However, the beam oscillation can modify the criterion of the welding mode transformation and change the weld formation mechanisms, which further leads to unstable control of penetration and affects welding quality.

Good penetration is conductive to ensuring product quality [15,16,17]. However, the relationship between the welding process parameters and the welding depth (WD) is unknown, non-linear, and complicated, thereby rendering it impractical to determine the optimal process parameters intuitively, even for skilled operators. Moreover, the beam oscillation welding increased process parameters and made the weld process more complicated [18,19,20].

In response to this problem, scholars from various countries have conducted numerous experimental studies, mainly to study the effect of different oscillation parameters on the WD for optimizing the welding process parameters and improving the welding methods and equipment. Li et al. established that the WD decreased significantly as the oscillation diameter and frequency increased, and the weld penetration in circular oscillating laser welding for the 5083 aluminum alloy is approximately proportional to the line energy of laser oscillation [21]. Li et al. found that when the welding oscillation amplitude of 304SS was lower than 0.4 mm, the weld penetration was unchanged; otherwise, the weld penetration and area were greatly decreased as the oscillation amplitude increased, an outcome caused by the welding mode variation [3]. Chen et al. concluded that the penetration of the “∞”-shaped laser scanning weld were inversely proportional to the scanning amplitude and scanning frequency of 5052 aluminum alloy, and the weld cross-section was a wide U-shape [7]. However, the relationship model between laser scanning welding parameters and penetration, especially for “∞”-shaped laser scanning welding, remains unexplored.

Although developed mathematical models such as polynomial response surface and radial basis function can be used to build the relationship between process parameters and welding seam geometry, the prediction performance of models cannot be guaranteed, even with numerous sample points [22,23,24], because of the coupling effect of welding parameters and the non-linear causality.

The excellent non-linear processing ability of the neural network has obvious advantages for predicting weld forming and mechanical properties [25,26]. However, the mathematical principle of a BP neural network determines its inherent defect of easily falling into the local optimum. In addition, its training effect is too dependent on the initial random weights and thresholds [17]. Compared with traditional neural network methods, the neuroevolution of augmenting topologies (NEAT) creates artificial neural networks through simulated evolution and allows the network topology and the connection weights to be simultaneously optimized through an evolutionary process [27]. Moreover, the NEAT searches through a minimal number of weight dimensions, thereby significantly reducing the number of generations necessary to identify a solution [28].

This work builds an “∞”-shaped laser scanning welding experimental platform based on a motion controller and a galvanometer scanner control gateway that are self-developed, takes the autogenous bead-on-plate welding of 304SS with 3 mm thick specimens as the experimental objects, and focuses on a model based on the neuroevolution of augmenting topologies for predicting the WD of “∞”-shaped laser scanning welding, in which input parameters synthesize laser power (LP), welding speed (WS), scanning frequency (SF), and scanning amplitude (SA). To solve the problem in which the distribution characteristics of the original sample data cannot be accurately described even with numerous sample points, the Latin hypercube sampling (LHS) is introduced in the design of the experimental parameters.

The verification experiment confirms that the prediction accuracy can meet the requirements of the main process parameter determination and subsequent penetration control. The model can accurately reflect the nonlinear relationship between the main welding parameters and WD, a feature which is helpful to ensure the penetration quality of “∞”-shaped laser scanning welding.

## 2. Experimental Setup

### 2.1. Experimental Platform

**Figure 1:**
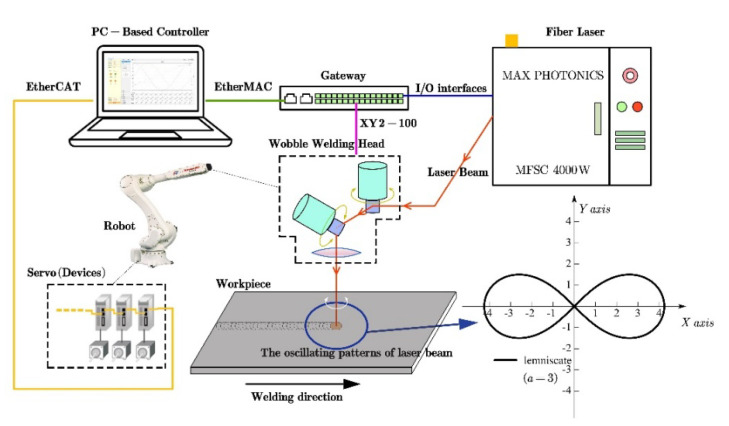
Scheme of the experimental setup for laser scanning welding.

The experimental setup (Figure 1) included a MAX PHOTONICS MFSC-4000 fiber laser (Maxphotonics Co., Ltd., Shenzhen, China), an OSPRI LDW400 wobble welding head (OSPRI Co., Ltd., Shenzhen, China), a Kawasaki six-axis industrial robot, a PC-based motion controller (self-developed), and a galvanometer scanner control gateway (self-developed). This fiber laser was a continuous beam mode with a wavelength of 1070 nm and a beam parameter product (BPP) of 2.8 mm mrad. The radius of the focused laser beam spot was approximately 0.4 mm. The wobble welding head consisted of a collimation unit with a focal length of 75 mm, two galvanometer scanner units, and a focusing unit with a focal length 300 mm. The beam oscillation was controlled by the galvanometer scanner and can be oscillated up to a maximum frequency of 250 Hz. The beam diameter is 1.6 mm at the focal position. The welding head was driven by the robot to move linearly in the X-direction.

**Figure 2:**
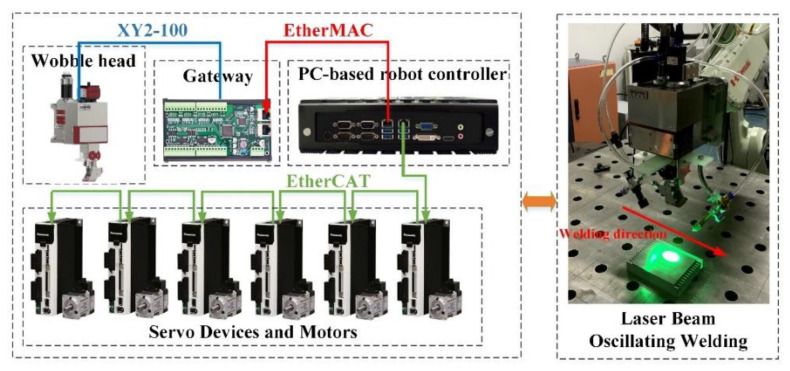
The control system layout.

The control system layout is shown in Figure 2. The experiment is implemented in a self-developed, PC-based motion controller with an Intel(R) Core (TM) i5-7200 U 2.5 GHz CPU, 16.00 GB SDRAM computer (Shandong Ezcode Intelligent Technology Co., Ltd., Jinan, China) and a Windows 10 operating system extended by Kithara real-time suite (KRTS) to guarantee real-time performance [29].

The PC-based motion controller acts as the master node on the EtherCAT Indus-tral Ethernet Network (Beckhoff Automation GmbH & Co. KG, Verl, Germany) and EtherMAC Industrial Ethernet Network (Shandong Ezcode Intelligent Techology Co. Ltd., Jinan, China) [30]. On the one hand, the motion controller communicates with the corresponding robot servo drives by the EtherCAT Industrial Ethernet Network, where the communication period is set to 1 ms, and the robot servo drives act as the slave node on the EtherCAT network. On the other hand, galvanometer scanner units that are in the OSPRI wobble welding head connect to the EtherMAC Industrial Ethernet Network via the self-developed galvanometer scanner control gateway module with Altera EP4CE6E22C8 FPGA (Altera Corporation., California, USA), where the com-munication period is set to 250 us and the module acts as a slave EtherMAC node. Moreover, the galvanometer scanner control gateway module communicates with the galvanometer scanner units via the XY2-100 serial buses, where the communication period is set to 10 us. The “one-transmission-multiple-conversion” manner is used to align the communication period, the gateway module receives a network frame once, and issues the instructions in multiple sequential conversions.

Based on the KRTS, the operating system of the motion controller can be divided into two sub-systems: the non-real-time operating system (non-RTOS) which can con-duct the tasks with no real-time requirements, and the KRTS-Kernel which is a re-al-time system with excellent real-time performance. The real-time interpolation stage of the robot and galvanometer scanner units are conducted in the KRTS-kernel, and the HMI task can be implemented in non-RTOS.

### 2.2. Experimental Procedure

The experiments were conducted using 3-mm-thick 304SS plates (in rolled and annealed condition) measuring 120 × 50 mm^2^ as the base metal. The chemical composi-tion and mechanical properties are listed in Table 1. During welding, the weld surface was protected by gas nozzles using pure argon. The gas flow of the nozzles was 15 L/min. Before the experiment, the surfaces of the plates were washed with acetone. After the plates were dried, the surface oxide film was scraped off with sandpaper, and the plates were washed with absolute ethanol.

**Figure 3:**
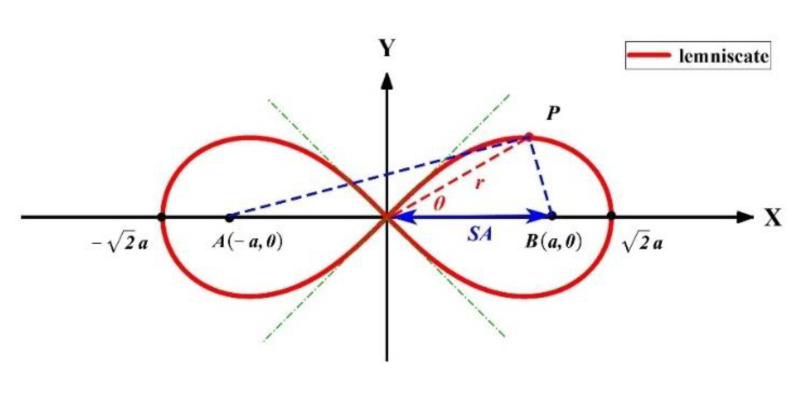
Schematic diagram of the “∞”-shaped curve.

**Figure 4:**
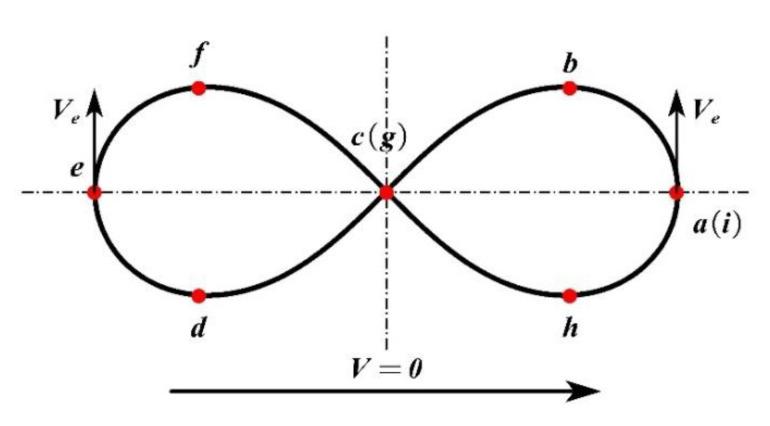
Static scanning trajectory of “∞”-shaped scanning welding.

The working principle of the wobble welding head is shown in Figure 1. In laser beam oscillation welding, the laser beam is collimated and then irradiated to the ro-tating mirrors. The deflection of the beam deflectors is driven by motors to achieve the scanning trajectory. The scanning pattern used is “∞”-shaped, also known as lemnis-cate. The “∞”-shaped curve is presented in Figure 3, the length of line *AB* is set to 2 *a*, if the moving point “*P*” satisfies Equation (1), and the trajectory of “P” is designated as the lemniscate:

(1) $$\left|PA\right|*\left|PB\right|=a^{2}$$

when the WS is *V* = 0, the trajectory of the laser beam is as shown in Figure 4. With point “*a*” as the starting point of a welding cycle, the laser beam is continuously circulated with scanning speed *Ve* along the path *a(i)*-*b*-*c(g)*-*d*-*e*-*f*-*g(c)*-*h*-*i(a)*.

**Figure 5:**
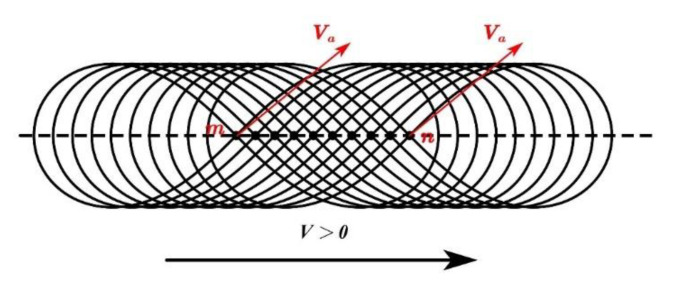
Dynamic continuous scanning trajectory of “∞”-shaped scanning welding.

The *Ve* is mainly determined by the scanning frequency (SF) and the scanning amplitude (SA) of the laser beam. The definition of the SA of the track is shown in Figure 3. When the WS is *V* > 0, the scanning trajectory of the laser is determined by the WS and the *Ve*. Figure 5 shows the continuous scanning trajectory of the “∞”-shaped motion of the laser beam.

The laser beam starts from point “*m*”. The welding process ends at point “*n*” after several motions. In the dynamic scanning process, the actual speed *Va* is a vector combination of the *Ve* and the *V* along the weld seam, which can be calculated using Equation (2). That is, the actual speed of “∞”-shaped scanning welding in the “*a(i)*-*b*-*c(g)*-*d*” and “*e*-*f*-*g(c)*-*h*-*i(a)*” segments is far higher than that of the single pass laser WS [7].

(2) $$\vec{V_{a}}=\vec{V_{e}}+\vec{V}$$

For obtaining different penetration morphology welded joints, autogenous bead-on-plate welding experiments were performed to investigate the effect of “∞”-shaped laser scanning welding parameters on welding penetration morphology. The LHS method was used to design the experimental parameters. This sampling method can accurately determine the design space by sampling with fewer iterations and distributing the sample points evenly throughout the design space. Generally, the welding depth of laser scanning welding is affected by laser power, welding speed, scanning pattern, gap, laser beam focal position, shielding gas, etc. These input factors commonly determine WD. Whether in conduction mode or keyhole mode, a nonlinear relationship between welding parameters and the welding depth is considered in this study. According to a large number of actual experiences, surveys, and primary investigations, the WD of “∞”-shaped laser scanning welding is mainly determined by four main factors, including LP, WS, SF, and SA, and high welding speed may lead to defects of incomplete fusion and even spatter, meanwhile high laser power may cause excessive penetration and dent [7,14]. Considering the actual conditions of the wobble welding head, the bounds of scanning parameters (SF and SA) are set, and the working ranges of all selected process parameters are fixed by conducting trail tests. In addition, considering the actual welding conditions and experimental costs, 60 samples of experimental parameters were determined by the LHS method. The working ranges of all selected experimental process parameters are as follows:

$$\begin{array}{l} 800\leq LP\leq 3000\;(W)\\ 8\leq WS\leq 30\;(mm/s)\\ 10\leq SF\leq 250\;(Hz)\\ 0\leq SA\leq 2.5\;(\mathrm{mm}) \end{array}$$

Other welding parameters are controlled as constant values, and other constant welding parameters in the experiment are shown in Table 2. After welding, the weldment is cut, polished, and etched along the section of the image sampling point by a wire cutting machine. Under the VMA video measuring machine, the macroscopic metallography of the fusion zone is observed and photographed to measure the relevant welding penetration characteristics.

## 3. Methodology and Model

### 3.1. Neuroevolution of Augmenting Topologies

By (1) employing a principled method of crossover of different topologies, (2) protecting structural innovation using speciation, and (3) incrementally growing from a minimal structure, the NEAT allows solutions to become incrementally more complex while they become more optimal [28].

**Figure 6:**
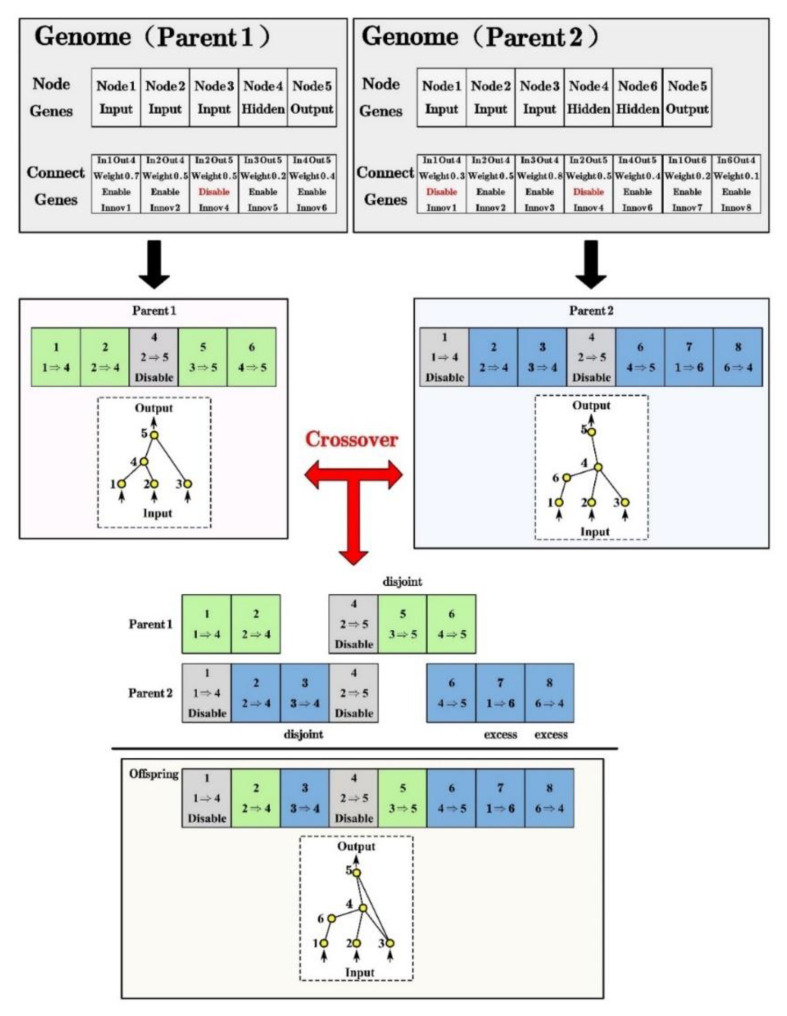
The genotype to phenotype mapping and matching up genomes for different network topologies.

As shown in Figure 6, each genome includes a list of connection genes, each of which refers to two node genes being connected in the NEAT. Each connection gene specifies the in-node, the out-node, the weight of the connection, whether or not the connection gene is expressed (an enable bit), and an innovation number (Innov), thereby facilitating the identification of corresponding genes during crossover. The innovation numbers represent a chronology of every gene in the system and can monitor the historical origin of every gene.

**Figure 7:**
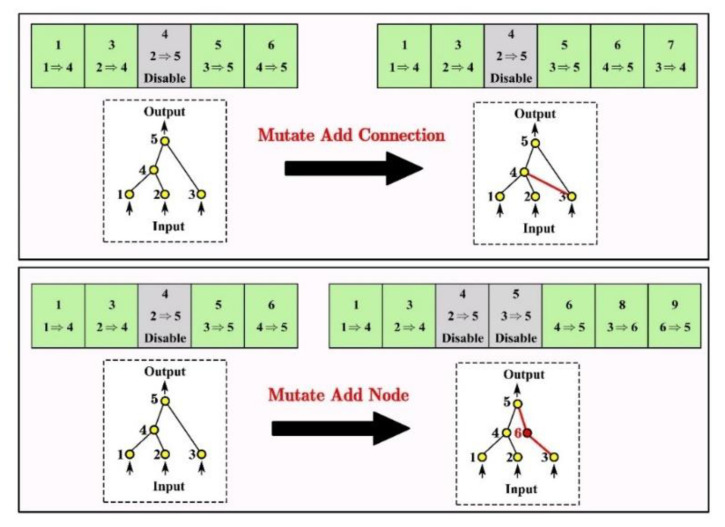
Two types of structural mutation in the NEAT.

A mutation in the NEAT can change not only connection weights, but also network structures. Structural mutations, which expand the genome, occur in two ways: connection mutations and node mutations (Figure 7). Through mutation, genomes of varying sizes are created, sometimes with completely different connections specified at the same positions.

When crossing over, the genes in both genomes with the same innovation numbers are lined up. Genes that do not match are inherited from the more fit parent, or if they are equally fit, from both parents randomly. Moreover, genes that do not match are either disjoint or excess, depending on whether they occur within or outside the range of the other parent’s innovation numbers in Figure 6.

The population is divided into species according to historical markings and topological similarity. The compatibility distance (φ) of different structures can be calculated using Equation (3).

(3) $$\varphi =\frac{a_{1}*E}{N}+\frac{a_{2}*D}{N}+a_{3}*\bar{W}$$

where *E* is the number of excess genes; *D* is the number of disjoint genes; $\bar{W}$ is the average weight difference of matching genes; *N* is the number of genes in the larger genome; and *a_1_*, *a_2_*, and *a_3_* are coefficients that adjust the importance of the three factors.

The NEAT speciates the population and shares fitness, so that individuals compete primarily within their own niches instead of against the entire population. Thus, individuals have time to optimize their structure before they must compete with other niches in the population, and this feature can effectively solve the problem of fitness reduction when adding new structures to a network.

Moreover, because the NEAT protects innovation using speciation, it can start this way minimally and grow new structures only as necessary. This way, the NEAT searches through a minimal number of weight dimensions, thereby significantly reducing the number of generations necessary to find a solution.

### 3.2. Establishment and Training of the NEAT Model

**Figure 8:**
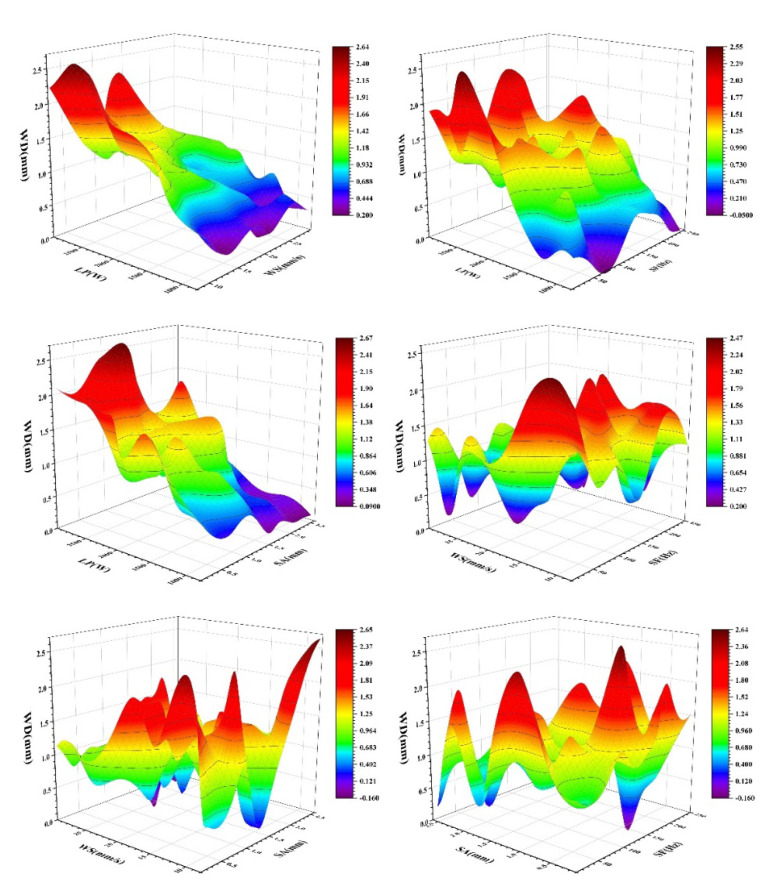
Welding depth changing with the process parameters.

A model was established and trained for describing the relationships between process parameters and WD. The experimental samples are demonstrated in Figure 8, and each graph demonstrates the changing WD with the process parameters.

**Figure 9:**
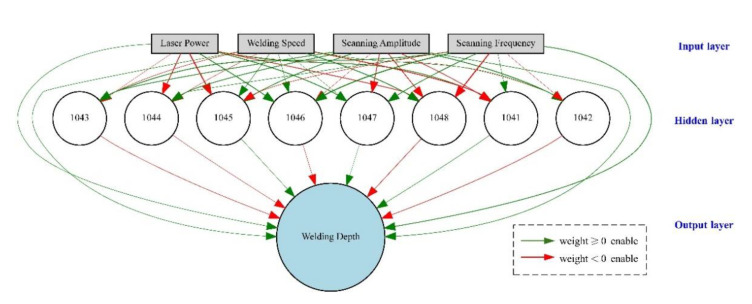
The initial topological structure of the model.

The procedure of establishing and training the model is planned in detail as follows:

1. Determination of the network model structure. The main goal is to predict the WD by selecting LP, WS, SF and SA as input parameters. Therefore, LP, WS, SF and SA are selected as the input parameters of the NEAT model, and the WD is taken as the output parameter. In the current related research, whether increasing the number of hidden layers can reduce the network error is uncertain but doing so will undoubtedly complicate the network structure and greatly increase the network training time and data occupation space. Therefore, a three-layer network with a single hidden layer and eight hidden layer nodes is selected in the initial design, the input layer nodes are connected to every node in the hidden layer and the output layer, every hidden layer node is connected to the output layer node, and the initial connections in the network are enabled. During training, the network topology and the connection weights are changed by crossing over and mutating to obtain the network structure with the smallest error. Consequently, the NEAT model for predicting WD is determined, and the initial topological structure of the model is shown in Figure 9, where the numbers in the hidden layer represent the innovation numbers, the thickness of connection lines are related to the value of initial random weights, the red lines mean weight < 0, the green lines mean weight ≥ 0, and the solid lines mean that the corresponding connections are enabled.
2. Design of training dataset and testing dataset. As mentioned, among 60 samples whose experimental parameters were determined by the LHS method, the experimental results of 58 samples involved incomplete penetration. The large difference of the different characteristic value of samples is not conducive to processing, so the samples are preprocessed through mean removal. Fifty samples (training dataset) are randomly selected for training the NEAT model from preprocessed samples. The remaining eight samples (testing dataset) were selected for testing prediction accuracy.
3. Set initial population and fitness rules. The initial population size is set to 300, and each individual in the population represents a network. The score of each individual is calculated according to the network calculation rules. To evaluate the quality of a solution, initial fitness minus the square of error between the output value and the expected value of training dataset is usually employed as the fitness. Thus, the higher the score, the smaller the proof error and the better the individual.
4. Implement the NEAT to obtain the prediction model. The NEAT was applied to solve the prediction problem and obtain the predicted value of WD. The program was developed based on Python 3.6 and was run in JetBrains PyCharm. Selected parameters for the NEAT are listed in Table 3.

## 4. Result and Discussion

### 4.1. Experimental Results

**Figure 10:**
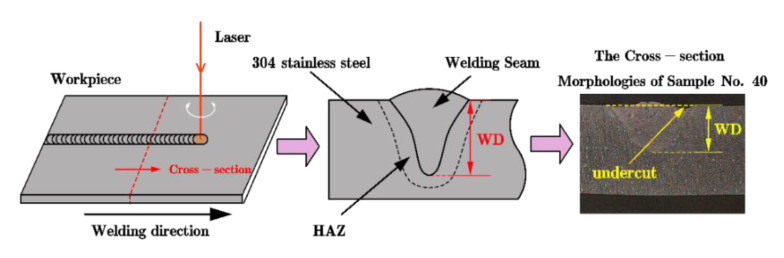
Geometrical features of the welding penetration morphology.

The WD is selected as the welding penetration depth evaluation index to evaluate the welding process in this work. The macroscopic metallographic diagram and dimensional measurement of the weldment sampling (No. 40) are shown in Figure 10.

**Figure 11:**
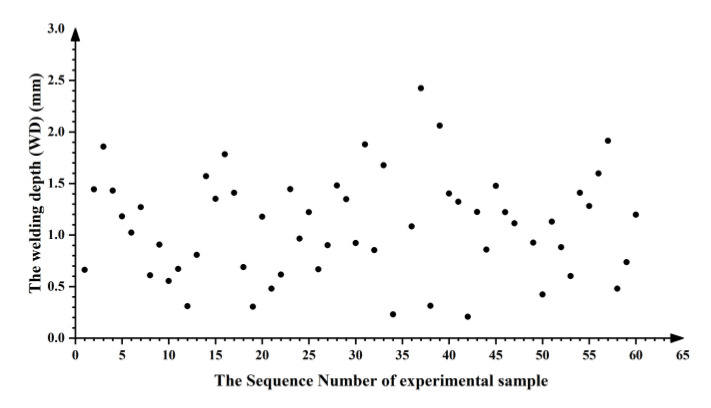
Welding depths of 58 experimental samples.

In Figure 11, the experimental results show that within the experimental range, the experimental samples 35 and 48 were completely penetrated, and the WD of the remaining 58 samples of experimental samples exhibit relatively uniform random distribution throughout the design space. Thus, the LHS method can accurately determine the design space by sampling with fewer iterations and describe the distribution characteristics of data. All experimental parameters and the measurement results of the samples are shown in Appendix A.

### 4.2. Analysis of the NEAT Model Training Process

**Figure 12:**
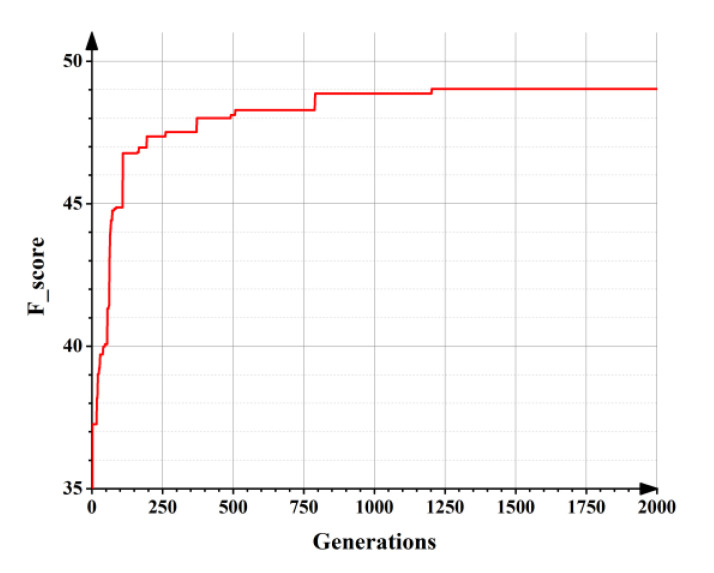
The best fitness of the population as the population evolves.

**Figure 13:**
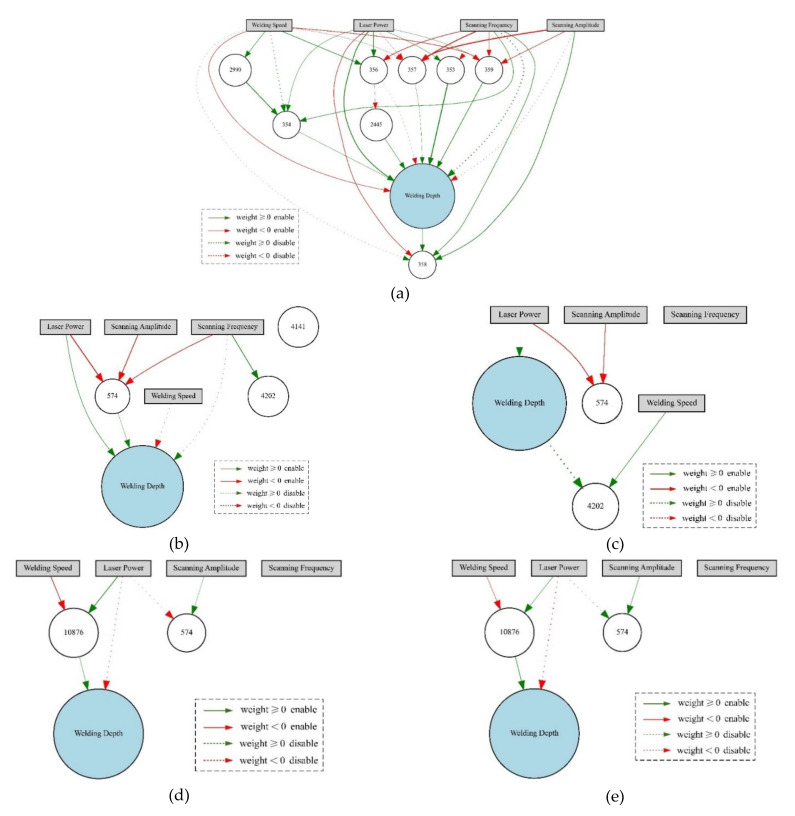

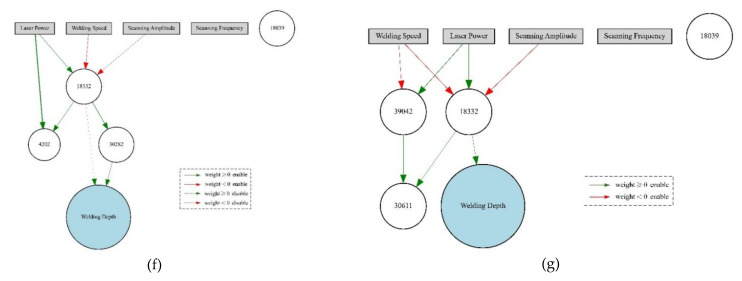
The topological structure of the model as the population evolves.

Each individual represents a network in the population and the fitness of each individual is calculated according to the network calculation rules and the initial fitness. The higher the fitness, the smaller the proof error and the better the individual. As the population iteration, the population goes through creation, selection, crossover, and mutation. In the process, individuals with high fitness are retained, while individuals with low fitness are eliminated. Therefore, the value of the best fitness (F_score) increass continuously (Figure 12), and the topological structure also evolves continuously (Figure 13). The topological structure of the NEAT model grows incrementally from a minimal structure, thereby significantly reducing the number of generations necessary to find a solution. Among the iterations, Figure 13a–g correspond to the topological structure of the NEAT model after 25, 50, 75, 175, 275, 525, and 800 generations, of evolution, respectively, and this outcome also means the prediction error decreases continuously.

**Figure 14:**
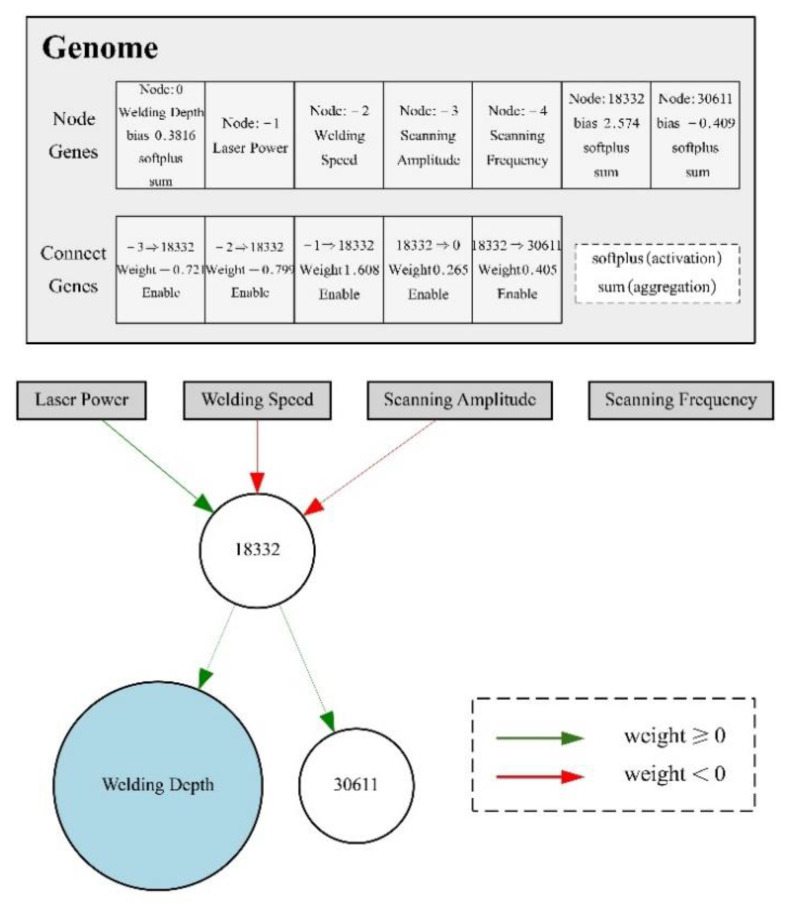
The final topological structure of the NEAT model.

The best individual gained by iterations is found and decoded, and the connection weights and network structures are obtained for the final network. The final network structure of the NEAT model is as shown in Figure 14. The weight of the connection and network topology are related to the effects of each process parameter on WD. In the working ranges of selected experimental process parameters, the main effects on WD include LP, WS, and SA. SF has almost no effect on WD. WD is proportional to LP and inversely proportional to WS and SA. The effect of every process parameter on the WD can be determined indirectly by predicting WD in the process parameters’ preliminary selection.

### 4.3. Validation of the Prediction Accuracy of the Proposed Approach

**Figure 15:**
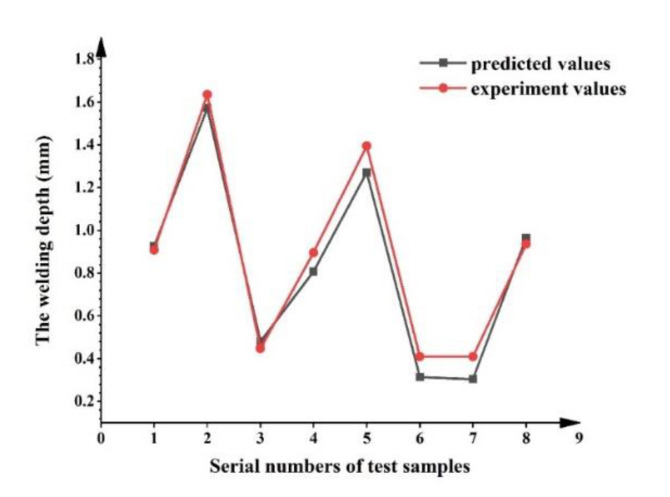
Comparison of predicted and experimental values.

The established NEAT model can describe the relationships between the process parameters and WD. To verify the prediction performance of the model, eight previously randomly selected samples (testing dataset) were used to predict WD. Comparative analysis of the predicted values and experiment values (Figure 15) show good agreement. The prediction accuracy is comparable to that of references [31,32], such that can meet the requirements of actual welding. To evaluate the performance of the model more intuitively, the NRMSE of the model was selected as the evaluation indicator. The NRMSE of WD is approximately 6.2%, an outcome that shows that the model has high prediction accuracy. Overall, the established NEAT model is reliable and can be used for predicting WD in the process parameter selection.

## 5. Conclusions

In this study, a “∞”-shaped laser scanning welding test platform based on a self-developed motion controller and galvanometer scanner control gateway is built. The experimental platform combined galvanometer scanners and robots over standard industrial ethernet networks, taking the autogenous bead-on-plate welding of 304SS with 3 mm thick specimens as the experiment objects and designing the experimental parameters by the LHS method. According to the experimental data, a neuroevolution of augmenting topologies (NEAT)-based model for predicting the penetration depth of the “∞”-shaped laser scanning welding is established. The results demonstrate that the model has good predictive performance and can provide reliable process guidance for “∞”-shaped laser scanning welding. The following main conclusions can be derived:

1. To some degree, the welding depth (WD) can represent the seam quality. The established NEAT model based on the main process parameters (laser power [LP], welding speed [WS], scanning amplitude [SA], and scanning frequency [SF]) as inputs and WD as output could accurately reflect the nonlinear relationship between the main welding parameters and WD, whether in conduction mode or in keyhole mode.
2. The NEAT model had high accuracy through verification tests and could predict the WD of the “∞”-shaped laser scanning welding results within acceptable error margins. Moreover, the normalized root mean square error (NRMSE) of WD is approximately 6.2% by validation.
3. Good prediction performance thus makes the model reliable for the preliminary selection of process parameters, and the proposed approach lays the foundation for controlling penetration and evaluating the quality of “∞”-shaped laser scanning welding. However, the welding depth is also influenced by other factors, even if their effect is usually limited. Therefore, follow-up research is needed before the application of this method in industry.

## Figures and Tables

**Table 1:** Chemical composition and mechanical properties of the 304SS plate.

C	Si	Mn	P	S	Cr	Ni	Cu	Fe
0.027	0.56	1.55	0.031	0.001	18.0	8.0	0.1	Bal.
Tensile strength/Mpa 660			Yield strength/Mpa 277			Elongation percentage/% 62.0		

**Table 2:** Other constant welding parameters.

Other Constant Welding Parameters	Value
The gas flow of the nozzles (L/min)	15
Defocusing distance (mm)	0
Plate thickness (mm)	3

**Table 3:** Selected parameters for the NEAT.

Parameter	Value
Fitness threshold	49.8
Activation_options	Softplus, Relu, Sigmoid
Activation_default	Softplus
Activation_mutate_rate	0.1
Aggregation_default	Sum
Conn_add_prob	0.5
Conn_delete_prob	0.5
Node_add_prob	0.2
Node_delete_prob	0.2
Enabled_default	True
Enabled_mutate_rate	0.05
Initial fitness	50
Population size	300
Maximum iterations	2000
Num_hidden	8
Num_inputs	4
Num_outputs	1
Initial_connection	Full_direct
Compatibility_disjoint_coefficient	1.0
Compatibility_weight_coefficient	0.5
Compatibility_threshold	3.0
Elitism	3

## Appendix A

**Table A1:** Experimental parameters and measurement results of 60 samples.

NO.	LP (W)	WS (mm/s)	SF (Hz)	SA (mm)	WD (mm)
1	1089	21	161	0.4	0.66
2	2228	13.2	197	0.8	1.44
3	2528	12.7	129	0.4	1.86
4	1759	10.4	79	0.6	1.43
5	1529	14.8	106	0.4	1.18
6	1740	28	25	0.4	1.02
7	2944	23.9	43	1.6	1.27
8	1624	28.8	120	1.3	0.61
9	2280	25.9	88	1.4	0.90
10	1387	25.3	236	1.1	0.56
11	1261	21.8	93	0.8	0.67
12	1036	13.8	247	1.6	0.31
13	2255	23.5	179	1.7	0.81
14	2438	13	228	1	1.57
15	2788	16.3	111	1.3	1.35
16	2031	8.8	204	0.1	1.79
17	1684	18.4	30	0	1.41
18	1129	15.3	66	0.9	0.69
19	1223	26.8	22	1.8	0.30
20	2654	17.3	163	1.8	1.18
21	962	27.5	18	1.2	0.48
22	863	9.8	139	0.8	0.62
23	2179	15.6	84	0.6	1.44
24	1846	26.2	167	0.6	0.97
25	2366	22.2	125	0.8	1.22
26	1454	27.4	136	1	0.67
27	1569	19.9	222	1	0.90
28	1990	14.6	172	0.1	1.48
29	1316	8.6	97	0.1	1.35
30	1489	18.9	145	1.2	0.92
31	2686	11.1	36	1.6	1.88
32	1825	19.1	221	1.4	0.85
33	2494	14	39	0.9	1.68
34	895	21.4	218	1.1	0.23
35	2972	12.2	54	0.1	Whole
36	1196	8	53	0.7	1.08
37	2475	9.9	14	0.7	2.43
38	1144	29.2	77	1.5	0.31
39	2823	17	183	0.1	2.06
40	2899	22.8	198	1.3	1.40
41	2379	20.6	243	0.6	1.32
42	930	20.1	61	1.5	0.21
43	1879	16	99	0.5	1.22
44	2047	22.5	188	1.6	0.86
45	2142	10.8	115	1.1	1.48
46	2877	24.8	46	1.6	1.22
47	1910	17.9	153	0.8	1.11
48	2743	12	146	0.5	Whole
49	1965	29.5	103	0.6	0.93
50	820	19.6	190	0.5	0.42
51	2106	28.2	131	0.2	1.13
52	2319	24.5	63	1.7	0.88
53	1007	26.7	212	0.3	0.60
54	2610	17.8	207	1.1	1.41
55	2572	25.1	27	1.1	1.28
56	1599	9.4	175	0.2	1.60
57	2747	16.8	157	0.4	1.92
58	1311	23.4	232	1.3	0.48
59	1353	29.8	70	0.3	0.74
60	1672	11.4	241	1.3	1.20
